# [(Nitrato-κ^2^ *O*,*O*′)(nitrito-κ^2^ *O*,*O*′)(0.25/1.75)]bis­(1,10-phenanthroline-κ^2^ *N*,*N*′)cadmium(II)

**DOI:** 10.1107/S1600536811002431

**Published:** 2011-01-22

**Authors:** Ezzatollah Najafi, Mostafa M. Amini, Seik Weng Ng

**Affiliations:** a Department of Chemistry, General Campus, Shahid Beheshti University, Tehran 1983963113, Iran; b Department of Chemistry, University of Malaya, 50603 Kuala Lumpur, Malaysia

## Abstract

The reaction of cadmium nitrate and sodium nitrite in the presence of 1,10-phenanthroline yields the mixed nitrate–nitrite title complex, [Cd(NO_2_)_1.75_(NO_3_)_0.25_(C_12_H_8_N_2_)_2_]. The metal ion is bis-chelated by two *N*-heterocycles as well as by the nitrate/nitrite ions in a distorted dodeca­hedral CdN_4_O_4_ coordination environment. One nitrite group is ordered; the other is disordered with respect to a nitrate group (ratio 0.75:0.25) concerning the O atom that is not involved in bonding to the metal ion.

## Related literature

For the crystal structure of [Cd(NO_3_)_2_(C_12_H_8_N_2_)_2_], see: Tadjarodi *et al.* (2001 ▶) and for the crystal structure of [Cd(NO_2_)_2_(C_12_H_8_N_2_)_2_], see: Abedini *et al.* (2005 ▶).
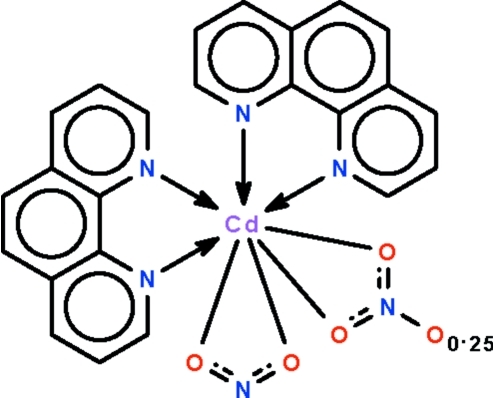


## Experimental

### 

#### Crystal data

[Cd(NO_2_)_1.75_(NO_3_)_0.25_(C_12_H_8_N_2_)_2_]
*M* *_r_* = 568.83
Triclinic, 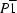

*a* = 9.1470 (4) Å
*b* = 10.1866 (4) Å
*c* = 13.0057 (6) Å
α = 76.953 (4)°
β = 77.270 (4)°
γ = 70.404 (4)°
*V* = 1098.27 (8) Å^3^
*Z* = 2
Mo *K*α radiation
μ = 1.04 mm^−1^
*T* = 100 K
0.30 × 0.20 × 0.10 mm

#### Data collection

Agilent Technologies SuperNova Dual diffractometer with an Atlas detector
Absorption correction: multi-scan (*CrysAlis PRO*; Agilent Technologies, 2010 ▶) *T*_min_ = 0.745, *T*_max_ = 0.903
8702 measured reflections
4852 independent reflections
4256 reflections with *I* > 2σ(*I*)
*R*_int_ = 0.032

#### Refinement

*R*[*F* ^2^ > 2σ(*F* ^2^)] = 0.033
*wR*(*F* ^2^) = 0.073
*S* = 1.02
4852 reflections
325 parameters
H-atom parameters constrained
Δρ_max_ = 0.49 e Å^−3^
Δρ_min_ = −0.69 e Å^−3^

### 

Data collection: *CrysAlis PRO* (Agilent Technologies, 2010 ▶); cell refinement: *CrysAlis PRO*; data reduction: *CrysAlis PRO*; program(s) used to solve structure: *SHELXS97* (Sheldrick, 2008 ▶); program(s) used to refine structure: *SHELXL97* (Sheldrick, 2008 ▶); molecular graphics: *X-SEED* (Barbour, 2001 ▶); software used to prepare material for publication: *publCIF* (Westrip, 2010 ▶).

## Supplementary Material

Crystal structure: contains datablocks global, I. DOI: 10.1107/S1600536811002431/wm2452sup1.cif

Structure factors: contains datablocks I. DOI: 10.1107/S1600536811002431/wm2452Isup2.hkl

Additional supplementary materials:  crystallographic information; 3D view; checkCIF report

## Figures and Tables

**Table 1:** Selected bond lengths (Å)

Cd1—O3	2.355 (2)
Cd1—N6	2.390 (2)
Cd1—N4	2.393 (2)
Cd1—N3	2.418 (2)
Cd1—O1	2.4547 (19)
Cd1—O4	2.503 (2)
Cd1—O2	2.5041 (19)
Cd1—N5	2.510 (2)

**Fig. 1.**
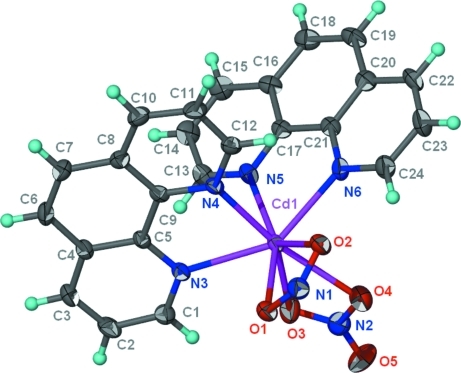
Thermal ellipsoid plot (Barbour, 2001) of Cd(NO2)1.75(NO3)0.25(C12H8N2)2 at the 70% probability level; hydrogen atoms are drawn as spheres of arbitrary radius.

## supplementary crystallographic information

### Comment

We had previously reported the structure of the 1,10-phenanthroline adduct of cadmium nitrate. In the corresponding structure, the cadmium ion, situated on a twofold rotation axis, shows eightfold coordination, which is somewhat less common (Tadjarodi *et al.*, 2001). The compound is conveniently synthesized by the direct addition of 1,10-phenanthroline to a cadmium nitrate solution. In a similar reaction, but when nitrite ions present, a mixed nitrate/nitrite compound is obtained.

In the title compound, Cd(NO_2_)_1.75_(NO_3_)_0.25_(C_12_H_8_N_2_)_2_ (Scheme I), the metal ion also exists in an eight-coordinate distorted dodecahedral CdN_4_O_4_ geometry (Fig. 1). The metal ion is bis-chelated by two *N*-heterocycles as well as by the nitrate/nitrite ions. The molecule lies on a general position, and one nitrite group is disordered with respect to a nitrate group (ratio 0.75:0.25).

### Experimental

Cadmium nitrate (1 mmol), sodium nitrite (1 mmol) and 1,10-phenanthroline (1 mmol) were loaded into a convection tube. The tube was filled with dry methanol and kept at 333 K. Colorless crystals were collected from the side arm of the tube after several days.

### Refinement

Carbon-bound H-atoms were placed in calculated positions [C—H 0.95 Å, *U*_iso_(H) 1.2*U*_eq_(C)] and were included in the refinement in the riding model approximation.

The structure, when refined as a dinitrite, had a high remaining peak approximately 1.2 Å away from one of the two N atoms of the nitrite groups. This site was allow to refine as an O atom of a disordered nitrate group. As the occupancy refined to nearly 1/4, its occupancy was eventually fixed as 0.25.

### Crystal data

**Table:**

[Cd(NO_2_)_1.75_(NO_3_)_0.25_(C_12_H_8_N_2_)_2_]	*Z* = 2
*M**_r_* = 568.83	*F*(000) = 568
Triclinic, *P*1	*D*_x_ = 1.720 Mg m^−^^3^
Hall symbol: -P 1	Mo *K*α radiation, λ = 0.71073 Å
*a* = 9.1470 (4) Å	Cell parameters from 4883 reflections
*b* = 10.1866 (4) Å	θ = 2.4–29.3°
*c* = 13.0057 (6) Å	µ = 1.04 mm^−^^1^
α = 76.953 (4)°	*T* = 100 K
β = 77.270 (4)°	Irregular, colorless
γ = 70.404 (4)°	0.30 × 0.20 × 0.10 mm
*V* = 1098.27 (8) Å^3^	

### Data collection

**Table:**

Agilent Technologies SuperNova Dual diffractometer with an Atlas detector	4852 independent reflections
Radiation source: SuperNova X-ray Source	4256 reflections with *I* > 2σ(*I*)
Mirror	*R*_int_ = 0.032
Detector resolution: 10.4041 pixels mm^-1^	θ_max_ = 27.5°, θ_min_ = 2.4°
ω scans	*h* = −10→11
Absorption correction: multi-scan (*CrysAlis PRO*; Agilent Technologies, 2010)	*k* = −13→11
*T*_min_ = 0.745, *T*_max_ = 0.903	*l* = −16→12
8702 measured reflections	

### Refinement

**Table:**

Refinement on *F*^2^	Primary atom site location: structure-invariant direct methods
Least-squares matrix: full	Secondary atom site location: difference Fourier map
*R*[*F*^2^ > 2σ(*F*^2^)] = 0.033	Hydrogen site location: inferred from neighbouring sites
*wR*(*F*^2^) = 0.073	H-atom parameters constrained
*S* = 1.02	*w* = 1/[σ^2^(*F*_o_^2^) + (0.0283*P*)^2^ + 0.1342*P*] where *P* = (*F*_o_^2^ + 2*F*_c_^2^)/3
4852 reflections	(Δ/σ)_max_ = 0.001
325 parameters	Δρ_max_ = 0.49 e Å^−^^3^
0 restraints	Δρ_min_ = −0.69 e Å^−^^3^

### Special details

**Table:**

Geometry. All e.s.d.'s (except the e.s.d. in the dihedral angle between two l.s. planes) are estimated using the full covariance matrix. The cell e.s.d.'s are taken into account individually in the estimation of e.s.d.'s in distances, angles and torsion angles; correlations between e.s.d.'s in cell parameters are only used when they are defined by crystal symmetry. An approximate (isotropic) treatment of cell e.s.d.'s is used for estimating e.s.d.'s involving l.s. planes.

### Fractional atomic coordinates and isotropic or equivalent isotropic displacement parameters (Å^2^)

**Table:**

	*x*	*y*	*z*	*U*_iso_*/*U*_eq_	Occ. (<1)
Cd1	0.55052 (2)	0.243966 (18)	0.241633 (15)	0.01614 (7)	
N1	0.7446 (3)	−0.0427 (2)	0.3117 (2)	0.0230 (5)	
N2	0.8042 (3)	0.3662 (3)	0.1701 (2)	0.0297 (6)	
N3	0.4863 (3)	0.2130 (2)	0.07978 (17)	0.0169 (5)	
N4	0.3528 (3)	0.1267 (2)	0.28117 (17)	0.0154 (5)	
N5	0.3074 (3)	0.4518 (2)	0.22889 (17)	0.0178 (5)	
N6	0.4512 (3)	0.3368 (2)	0.40496 (18)	0.0192 (5)	
O1	0.7414 (2)	0.01355 (19)	0.21528 (15)	0.0232 (4)	
O2	0.6588 (2)	0.0353 (2)	0.37712 (15)	0.0244 (5)	
O3	0.6973 (3)	0.3783 (2)	0.11795 (17)	0.0324 (5)	
O4	0.7905 (3)	0.2957 (2)	0.26214 (17)	0.0311 (5)	
O5	0.9091 (12)	0.4103 (10)	0.1371 (8)	0.042 (2)	0.25
C1	0.5549 (4)	0.2514 (3)	−0.0180 (2)	0.0207 (6)	
H1	0.6401	0.2882	−0.0259	0.025*	
C2	0.5079 (4)	0.2403 (3)	−0.1101 (2)	0.0245 (7)	
H2	0.5599	0.2697	−0.1788	0.029*	
C3	0.3864 (4)	0.1866 (3)	−0.1000 (2)	0.0214 (6)	
H3	0.3534	0.1778	−0.1617	0.026*	
C4	0.3102 (3)	0.1446 (3)	0.0025 (2)	0.0172 (6)	
C5	0.3661 (3)	0.1595 (2)	0.0909 (2)	0.0150 (5)	
C6	0.1825 (3)	0.0874 (3)	0.0203 (2)	0.0212 (6)	
H6	0.1455	0.0768	−0.0392	0.025*	
C7	0.1132 (4)	0.0480 (3)	0.1201 (2)	0.0220 (6)	
H7	0.0260	0.0133	0.1297	0.026*	
C8	0.1691 (3)	0.0577 (3)	0.2120 (2)	0.0181 (6)	
C9	0.2942 (3)	0.1139 (2)	0.1978 (2)	0.0151 (6)	
C10	0.1059 (4)	0.0116 (3)	0.3171 (2)	0.0231 (6)	
H10	0.0202	−0.0261	0.3305	0.028*	
C11	0.1695 (3)	0.0216 (3)	0.4007 (2)	0.0224 (6)	
H11	0.1302	−0.0116	0.4722	0.027*	
C12	0.2919 (3)	0.0807 (3)	0.3790 (2)	0.0193 (6)	
H12	0.3337	0.0884	0.4374	0.023*	
C13	0.2348 (4)	0.5050 (3)	0.1442 (2)	0.0208 (6)	
H13	0.2824	0.4683	0.0798	0.025*	
C14	0.0912 (4)	0.6131 (3)	0.1453 (2)	0.0242 (7)	
H14	0.0419	0.6469	0.0833	0.029*	
C15	0.0235 (4)	0.6689 (3)	0.2363 (2)	0.0246 (7)	
H15	−0.0732	0.7429	0.2381	0.029*	
C16	0.0972 (3)	0.6166 (3)	0.3281 (2)	0.0195 (6)	
C17	0.2391 (3)	0.5055 (3)	0.3202 (2)	0.0171 (6)	
C18	0.0342 (4)	0.6698 (3)	0.4263 (2)	0.0240 (7)	
H18	−0.0612	0.7451	0.4309	0.029*	
C19	0.1074 (4)	0.6156 (3)	0.5130 (2)	0.0240 (7)	
H19	0.0644	0.6547	0.5768	0.029*	
C20	0.2491 (4)	0.4999 (3)	0.5094 (2)	0.0209 (6)	
C21	0.3163 (3)	0.4449 (3)	0.4137 (2)	0.0175 (6)	
C22	0.3269 (4)	0.4374 (3)	0.5986 (2)	0.0242 (7)	
H22	0.2855	0.4706	0.6648	0.029*	
C23	0.4628 (4)	0.3281 (3)	0.5883 (2)	0.0269 (7)	
H23	0.5165	0.2837	0.6477	0.032*	
C24	0.5216 (4)	0.2824 (3)	0.4898 (2)	0.0233 (6)	
H24	0.6177	0.2080	0.4836	0.028*	

### Atomic displacement parameters (Å^2^)

**Table:**

	*U*^11^	*U*^22^	*U*^33^	*U*^12^	*U*^13^	*U*^23^
Cd1	0.01776 (13)	0.01855 (11)	0.01235 (11)	−0.00468 (8)	−0.00285 (8)	−0.00377 (8)
N1	0.0219 (14)	0.0227 (12)	0.0237 (14)	−0.0033 (10)	−0.0042 (11)	−0.0069 (11)
N2	0.0284 (17)	0.0363 (15)	0.0304 (15)	−0.0163 (13)	0.0004 (13)	−0.0124 (12)
N3	0.0190 (13)	0.0177 (11)	0.0145 (12)	−0.0060 (9)	−0.0005 (10)	−0.0050 (9)
N4	0.0149 (13)	0.0173 (11)	0.0116 (11)	−0.0024 (9)	−0.0016 (10)	−0.0021 (9)
N5	0.0247 (14)	0.0156 (11)	0.0137 (12)	−0.0073 (10)	−0.0027 (10)	−0.0021 (9)
N6	0.0219 (14)	0.0186 (11)	0.0189 (12)	−0.0043 (10)	−0.0082 (11)	−0.0043 (10)
O1	0.0218 (12)	0.0298 (10)	0.0171 (11)	−0.0051 (9)	−0.0027 (9)	−0.0063 (9)
O2	0.0224 (12)	0.0309 (11)	0.0177 (11)	−0.0022 (9)	−0.0037 (9)	−0.0081 (9)
O3	0.0370 (15)	0.0430 (13)	0.0277 (12)	−0.0244 (11)	−0.0071 (11)	−0.0057 (10)
O4	0.0328 (14)	0.0357 (12)	0.0275 (13)	−0.0096 (10)	−0.0054 (11)	−0.0110 (10)
O5	0.031 (6)	0.053 (6)	0.054 (6)	−0.035 (5)	0.001 (5)	−0.009 (5)
C1	0.0216 (17)	0.0242 (14)	0.0180 (15)	−0.0102 (12)	−0.0011 (12)	−0.0035 (12)
C2	0.0307 (19)	0.0293 (15)	0.0130 (14)	−0.0130 (13)	0.0029 (13)	−0.0027 (12)
C3	0.0274 (18)	0.0234 (14)	0.0154 (14)	−0.0083 (12)	−0.0044 (13)	−0.0056 (12)
C4	0.0192 (16)	0.0164 (13)	0.0161 (14)	−0.0030 (11)	−0.0045 (12)	−0.0048 (11)
C5	0.0160 (15)	0.0132 (12)	0.0140 (13)	−0.0003 (10)	−0.0034 (11)	−0.0043 (10)
C6	0.0227 (17)	0.0243 (14)	0.0201 (15)	−0.0068 (12)	−0.0091 (13)	−0.0056 (12)
C7	0.0182 (16)	0.0262 (14)	0.0248 (16)	−0.0086 (12)	−0.0045 (13)	−0.0062 (13)
C8	0.0148 (15)	0.0194 (13)	0.0188 (14)	−0.0032 (11)	−0.0024 (12)	−0.0038 (11)
C9	0.0148 (15)	0.0119 (12)	0.0143 (13)	0.0020 (10)	−0.0024 (11)	−0.0026 (10)
C10	0.0166 (16)	0.0298 (15)	0.0225 (16)	−0.0090 (12)	−0.0013 (13)	−0.0021 (13)
C11	0.0176 (16)	0.0305 (15)	0.0147 (14)	−0.0076 (12)	0.0008 (12)	0.0021 (12)
C12	0.0162 (15)	0.0235 (14)	0.0151 (14)	−0.0032 (11)	−0.0011 (12)	−0.0027 (11)
C13	0.0279 (18)	0.0167 (13)	0.0167 (14)	−0.0053 (12)	−0.0049 (13)	−0.0012 (11)
C14	0.0268 (18)	0.0201 (14)	0.0212 (16)	−0.0029 (12)	−0.0088 (14)	0.0038 (12)
C15	0.0252 (18)	0.0171 (13)	0.0278 (17)	−0.0012 (12)	−0.0071 (14)	−0.0015 (12)
C16	0.0213 (17)	0.0152 (13)	0.0188 (15)	−0.0046 (11)	0.0010 (12)	−0.0020 (11)
C17	0.0195 (16)	0.0157 (13)	0.0165 (14)	−0.0073 (11)	0.0004 (12)	−0.0032 (11)
C18	0.0243 (17)	0.0176 (13)	0.0275 (17)	−0.0053 (12)	0.0013 (14)	−0.0053 (12)
C19	0.0297 (18)	0.0203 (14)	0.0213 (16)	−0.0088 (13)	0.0046 (13)	−0.0088 (12)
C20	0.0231 (17)	0.0207 (14)	0.0205 (15)	−0.0101 (12)	−0.0005 (13)	−0.0038 (12)
C21	0.0214 (16)	0.0161 (13)	0.0155 (14)	−0.0086 (11)	−0.0007 (12)	−0.0014 (11)
C22	0.0327 (19)	0.0289 (15)	0.0147 (14)	−0.0134 (14)	0.0005 (13)	−0.0087 (12)
C23	0.035 (2)	0.0279 (15)	0.0201 (16)	−0.0068 (14)	−0.0116 (14)	−0.0051 (13)
C24	0.0240 (17)	0.0238 (14)	0.0226 (16)	−0.0036 (12)	−0.0077 (13)	−0.0064 (12)

### Geometric parameters (Å, °)

**Table:**

Cd1—O3	2.355 (2)	C6—H6	0.9500
Cd1—N6	2.390 (2)	C7—C8	1.434 (4)
Cd1—N4	2.393 (2)	C7—H7	0.9500
Cd1—N3	2.418 (2)	C8—C10	1.403 (4)
Cd1—O1	2.4547 (19)	C8—C9	1.403 (4)
Cd1—O4	2.503 (2)	C10—C11	1.377 (4)
Cd1—O2	2.5041 (19)	C10—H10	0.9500
Cd1—N5	2.510 (2)	C11—C12	1.391 (4)
N1—O2	1.250 (3)	C11—H11	0.9500
N1—O1	1.258 (3)	C12—H12	0.9500
N2—O5	1.151 (9)	C13—C14	1.403 (4)
N2—O4	1.254 (3)	C13—H13	0.9500
N2—O3	1.267 (3)	C14—C15	1.362 (4)
N3—C1	1.323 (3)	C14—H14	0.9500
N3—C5	1.349 (3)	C15—C16	1.410 (4)
N4—C12	1.320 (3)	C15—H15	0.9500
N4—C9	1.359 (3)	C16—C17	1.409 (4)
N5—C13	1.326 (3)	C16—C18	1.428 (4)
N5—C17	1.356 (3)	C17—C21	1.451 (4)
N6—C24	1.319 (3)	C18—C19	1.351 (4)
N6—C21	1.353 (4)	C18—H18	0.9500
C1—C2	1.398 (4)	C19—C20	1.430 (4)
C1—H1	0.9500	C19—H19	0.9500
C2—C3	1.365 (4)	C20—C22	1.409 (4)
C2—H2	0.9500	C20—C21	1.410 (4)
C3—C4	1.406 (4)	C22—C23	1.367 (4)
C3—H3	0.9500	C22—H22	0.9500
C4—C5	1.414 (3)	C23—C24	1.395 (4)
C4—C6	1.426 (4)	C23—H23	0.9500
C5—C9	1.447 (4)	C24—H24	0.9500
C6—C7	1.350 (4)		
			
O3—Cd1—N6	111.58 (7)	N3—C5—C4	122.6 (2)
O3—Cd1—N4	149.81 (7)	N3—C5—C9	118.3 (2)
N6—Cd1—N4	90.18 (8)	C4—C5—C9	119.1 (2)
O3—Cd1—N3	81.88 (7)	C7—C6—C4	121.2 (2)
N6—Cd1—N3	145.83 (8)	C7—C6—H6	119.4
N4—Cd1—N3	68.85 (7)	C4—C6—H6	119.4
O3—Cd1—O1	95.42 (7)	C6—C7—C8	121.0 (3)
N6—Cd1—O1	127.58 (7)	C6—C7—H7	119.5
N4—Cd1—O1	86.59 (7)	C8—C7—H7	119.5
N3—Cd1—O1	79.44 (7)	C10—C8—C9	117.5 (2)
O3—Cd1—O4	51.44 (7)	C10—C8—C7	123.1 (3)
N6—Cd1—O4	81.44 (8)	C9—C8—C7	119.4 (3)
N4—Cd1—O4	157.42 (7)	N4—C9—C8	122.6 (2)
N3—Cd1—O4	127.41 (8)	N4—C9—C5	117.8 (2)
O1—Cd1—O4	82.14 (7)	C8—C9—C5	119.6 (2)
O3—Cd1—O2	125.56 (7)	C11—C10—C8	119.2 (3)
N6—Cd1—O2	77.83 (7)	C11—C10—H10	120.4
N4—Cd1—O2	78.09 (7)	C8—C10—H10	120.4
N3—Cd1—O2	120.90 (6)	C10—C11—C12	119.3 (3)
O1—Cd1—O2	50.33 (6)	C10—C11—H11	120.4
O4—Cd1—O2	79.66 (7)	C12—C11—H11	120.4
O3—Cd1—N5	89.71 (8)	N4—C12—C11	123.0 (3)
N6—Cd1—N5	67.54 (7)	N4—C12—H12	118.5
N4—Cd1—N5	79.26 (7)	C11—C12—H12	118.5
N3—Cd1—N5	81.80 (7)	N5—C13—C14	123.1 (3)
O1—Cd1—N5	159.63 (7)	N5—C13—H13	118.4
O4—Cd1—N5	115.94 (7)	C14—C13—H13	118.4
O2—Cd1—N5	138.17 (7)	C15—C14—C13	119.1 (3)
O2—N1—O1	114.4 (2)	C15—C14—H14	120.5
O5—N2—O4	121.3 (5)	C13—C14—H14	120.5
O5—N2—O3	124.7 (6)	C14—C15—C16	119.8 (3)
O4—N2—O3	113.9 (2)	C14—C15—H15	120.1
C1—N3—C5	118.4 (2)	C16—C15—H15	120.1
C1—N3—Cd1	124.62 (18)	C17—C16—C15	117.0 (3)
C5—N3—Cd1	116.90 (17)	C17—C16—C18	119.7 (3)
C12—N4—C9	118.3 (2)	C15—C16—C18	123.2 (3)
C12—N4—Cd1	123.87 (17)	N5—C17—C16	122.9 (2)
C9—N4—Cd1	117.69 (17)	N5—C17—C21	118.1 (2)
C13—N5—C17	118.0 (2)	C16—C17—C21	119.0 (3)
C13—N5—Cd1	125.85 (19)	C19—C18—C16	121.5 (3)
C17—N5—Cd1	115.91 (17)	C19—C18—H18	119.3
C24—N6—C21	118.0 (3)	C16—C18—H18	119.3
C24—N6—Cd1	121.76 (19)	C18—C19—C20	120.5 (3)
C21—N6—Cd1	120.25 (17)	C18—C19—H19	119.7
N1—O1—Cd1	98.71 (15)	C20—C19—H19	119.7
N1—O2—Cd1	96.51 (15)	C22—C20—C21	117.6 (3)
N2—O3—Cd1	100.74 (17)	C22—C20—C19	122.5 (3)
N2—O4—Cd1	93.91 (16)	C21—C20—C19	119.9 (3)
N3—C1—C2	123.1 (3)	N6—C21—C20	122.6 (2)
N3—C1—H1	118.5	N6—C21—C17	118.0 (2)
C2—C1—H1	118.5	C20—C21—C17	119.3 (3)
C3—C2—C1	119.1 (3)	C23—C22—C20	119.0 (3)
C3—C2—H2	120.4	C23—C22—H22	120.5
C1—C2—H2	120.4	C20—C22—H22	120.5
C2—C3—C4	119.6 (2)	C22—C23—C24	119.3 (3)
C2—C3—H3	120.2	C22—C23—H23	120.4
C4—C3—H3	120.2	C24—C23—H23	120.4
C3—C4—C5	117.2 (2)	N6—C24—C23	123.5 (3)
C3—C4—C6	123.3 (2)	N6—C24—H24	118.2
C5—C4—C6	119.5 (3)	C23—C24—H24	118.2
			
O3—Cd1—N3—C1	9.7 (2)	O3—N2—O4—Cd1	1.6 (2)
N6—Cd1—N3—C1	126.5 (2)	O3—Cd1—O4—N2	−0.98 (16)
N4—Cd1—N3—C1	−177.9 (2)	N6—Cd1—O4—N2	−127.53 (17)
O1—Cd1—N3—C1	−87.4 (2)	N4—Cd1—O4—N2	163.19 (18)
O4—Cd1—N3—C1	−16.0 (2)	N3—Cd1—O4—N2	32.21 (19)
O2—Cd1—N3—C1	−117.2 (2)	O1—Cd1—O4—N2	102.42 (17)
N5—Cd1—N3—C1	100.5 (2)	O2—Cd1—O4—N2	153.38 (17)
O3—Cd1—N3—C5	−166.68 (19)	N5—Cd1—O4—N2	−67.72 (18)
N6—Cd1—N3—C5	−49.9 (2)	C5—N3—C1—C2	0.6 (4)
N4—Cd1—N3—C5	5.81 (17)	Cd1—N3—C1—C2	−175.7 (2)
O1—Cd1—N3—C5	96.22 (18)	N3—C1—C2—C3	−0.4 (4)
O4—Cd1—N3—C5	167.70 (16)	C1—C2—C3—C4	0.4 (4)
O2—Cd1—N3—C5	66.5 (2)	C2—C3—C4—C5	−0.5 (4)
N5—Cd1—N3—C5	−75.80 (18)	C2—C3—C4—C6	−179.9 (3)
O3—Cd1—N4—C12	−167.40 (19)	C1—N3—C5—C4	−0.7 (4)
N6—Cd1—N4—C12	−30.0 (2)	Cd1—N3—C5—C4	175.90 (19)
N3—Cd1—N4—C12	177.7 (2)	C1—N3—C5—C9	178.3 (2)
O1—Cd1—N4—C12	97.7 (2)	Cd1—N3—C5—C9	−5.2 (3)
O4—Cd1—N4—C12	37.7 (3)	C3—C4—C5—N3	0.7 (4)
O2—Cd1—N4—C12	47.6 (2)	C6—C4—C5—N3	−179.9 (2)
N5—Cd1—N4—C12	−97.0 (2)	C3—C4—C5—C9	−178.3 (2)
O3—Cd1—N4—C9	8.8 (3)	C6—C4—C5—C9	1.2 (4)
N6—Cd1—N4—C9	146.27 (18)	C3—C4—C6—C7	179.8 (3)
N3—Cd1—N4—C9	−6.09 (17)	C5—C4—C6—C7	0.4 (4)
O1—Cd1—N4—C9	−86.08 (18)	C4—C6—C7—C8	−2.2 (4)
O4—Cd1—N4—C9	−146.08 (19)	C6—C7—C8—C10	−176.7 (3)
O2—Cd1—N4—C9	−136.21 (19)	C6—C7—C8—C9	2.3 (4)
N5—Cd1—N4—C9	79.19 (18)	C12—N4—C9—C8	1.8 (4)
O3—Cd1—N5—C13	68.5 (2)	Cd1—N4—C9—C8	−174.59 (18)
N6—Cd1—N5—C13	−177.9 (2)	C12—N4—C9—C5	−177.6 (2)
N4—Cd1—N5—C13	−83.2 (2)	Cd1—N4—C9—C5	6.0 (3)
N3—Cd1—N5—C13	−13.3 (2)	C10—C8—C9—N4	−1.0 (4)
O1—Cd1—N5—C13	−36.4 (3)	C7—C8—C9—N4	179.8 (2)
O4—Cd1—N5—C13	114.5 (2)	C10—C8—C9—C5	178.4 (2)
O2—Cd1—N5—C13	−141.39 (19)	C7—C8—C9—C5	−0.7 (4)
O3—Cd1—N5—C17	−117.09 (18)	N3—C5—C9—N4	−0.5 (3)
N6—Cd1—N5—C17	−3.51 (17)	C4—C5—C9—N4	178.5 (2)
N4—Cd1—N5—C17	91.18 (19)	N3—C5—C9—C8	−179.9 (2)
N3—Cd1—N5—C17	161.08 (19)	C4—C5—C9—C8	−1.0 (4)
O1—Cd1—N5—C17	138.0 (2)	C9—C8—C10—C11	−0.8 (4)
O4—Cd1—N5—C17	−71.16 (19)	C7—C8—C10—C11	178.3 (3)
O2—Cd1—N5—C17	33.0 (2)	C8—C10—C11—C12	1.8 (4)
O3—Cd1—N6—C24	−99.0 (2)	C9—N4—C12—C11	−0.8 (4)
N4—Cd1—N6—C24	102.5 (2)	Cd1—N4—C12—C11	175.4 (2)
N3—Cd1—N6—C24	152.85 (19)	C10—C11—C12—N4	−1.0 (4)
O1—Cd1—N6—C24	16.6 (2)	C17—N5—C13—C14	−0.4 (4)
O4—Cd1—N6—C24	−56.5 (2)	Cd1—N5—C13—C14	173.9 (2)
O2—Cd1—N6—C24	24.7 (2)	N5—C13—C14—C15	1.4 (4)
N5—Cd1—N6—C24	−179.2 (2)	C13—C14—C15—C16	−0.7 (4)
O3—Cd1—N6—C21	83.4 (2)	C14—C15—C16—C17	−0.9 (4)
N4—Cd1—N6—C21	−75.1 (2)	C14—C15—C16—C18	179.7 (3)
N3—Cd1—N6—C21	−24.8 (3)	C13—N5—C17—C16	−1.4 (4)
O1—Cd1—N6—C21	−160.97 (17)	Cd1—N5—C17—C16	−176.21 (19)
O4—Cd1—N6—C21	125.9 (2)	C13—N5—C17—C21	178.5 (2)
O2—Cd1—N6—C21	−152.9 (2)	Cd1—N5—C17—C21	3.7 (3)
N5—Cd1—N6—C21	3.16 (18)	C15—C16—C17—N5	2.0 (4)
O2—N1—O1—Cd1	−1.1 (2)	C18—C16—C17—N5	−178.5 (2)
O3—Cd1—O1—N1	133.52 (16)	C15—C16—C17—C21	−177.9 (2)
N6—Cd1—O1—N1	10.90 (19)	C18—C16—C17—C21	1.6 (4)
N4—Cd1—O1—N1	−76.70 (16)	C17—C16—C18—C19	−0.3 (4)
N3—Cd1—O1—N1	−145.80 (16)	C15—C16—C18—C19	179.2 (3)
O4—Cd1—O1—N1	83.69 (16)	C16—C18—C19—C20	−1.6 (4)
O2—Cd1—O1—N1	0.64 (14)	C18—C19—C20—C22	−178.1 (3)
N5—Cd1—O1—N1	−122.5 (2)	C18—C19—C20—C21	2.2 (4)
O1—N1—O2—Cd1	1.1 (2)	C24—N6—C21—C20	−0.4 (4)
O3—Cd1—O2—N1	−64.39 (17)	Cd1—N6—C21—C20	177.34 (19)
N6—Cd1—O2—N1	−172.34 (17)	C24—N6—C21—C17	179.7 (2)
N4—Cd1—O2—N1	94.86 (16)	Cd1—N6—C21—C17	−2.6 (3)
N3—Cd1—O2—N1	38.65 (18)	C22—C20—C21—N6	−0.5 (4)
O1—Cd1—O2—N1	−0.64 (14)	C19—C20—C21—N6	179.1 (2)
O4—Cd1—O2—N1	−88.97 (16)	C22—C20—C21—C17	179.4 (2)
N5—Cd1—O2—N1	153.45 (15)	C19—C20—C21—C17	−0.9 (4)
O5—N2—O3—Cd1	175.7 (6)	N5—C17—C21—N6	−0.9 (4)
O4—N2—O3—Cd1	−1.7 (3)	C16—C17—C21—N6	179.0 (2)
N6—Cd1—O3—N2	59.66 (19)	N5—C17—C21—C20	179.1 (2)
N4—Cd1—O3—N2	−167.00 (16)	C16—C17—C21—C20	−1.0 (4)
N3—Cd1—O3—N2	−152.96 (18)	C21—C20—C22—C23	0.4 (4)
O1—Cd1—O3—N2	−74.47 (18)	C19—C20—C22—C23	−179.3 (3)
O4—Cd1—O3—N2	0.99 (16)	C20—C22—C23—C24	0.6 (4)
O2—Cd1—O3—N2	−30.6 (2)	C21—N6—C24—C23	1.5 (4)
N5—Cd1—O3—N2	125.28 (18)	Cd1—N6—C24—C23	−176.2 (2)
O5—N2—O4—Cd1	−175.9 (6)	C22—C23—C24—N6	−1.6 (5)
